# Targeted Gene Expression Profiling of Human Myeloid Cells From Blood and Lung Compartments of Patients With Tuberculosis and Other Lung Diseases

**DOI:** 10.3389/fimmu.2022.839747

**Published:** 2022-03-08

**Authors:** Leigh Ann Kotze, Gian van der Spuy, Bryan Leonard, Adam Penn-Nicholson, Munyaradzi Musvosvi, Shirley McAnda, Stephanus T. Malherbe, Mzwandile Erasmus, Thomas Scriba, Coenraad F. N. Koegelenberg, Brian W. Allwood, Gerhard Walzl, Nelita du Plessis

**Affiliations:** ^1^ DSI-NRF Centre of Excellence for Biomedical Tuberculosis Research, South African Medical Research Council Centre for Tuberculosis Research, Division of Molecular Biology and Human Genetics, Faculty of Medicine and Health Sciences, Stellenbosch University, Cape Town, South Africa; ^2^ South African Tuberculosis Vaccine Initiative, Division of Immunology, Department of Pathology, Institute of Infectious Disease and Molecular Medicine, University of Cape Town, Cape Town, South Africa; ^3^ Division of Pulmonology, Department of Medicine, Stellenbosch University and Tygerberg Academic Hospital, Cape Town, South Africa

**Keywords:** myeloid derived suppressor cells, alveolar macrophages, monocytes, tuberculosis, lung disease, bronchoalveolar lavage

## Abstract

Myeloid-derived suppressor cells (MDSC) have been identified in the peripheral blood and granulomas of patients with active TB disease, but their phenotype-, function-, and immunosuppressive mechanism- spectrum remains unclear. Importantly, the frequency and signaling pathways of MDSC at the site of disease is unknown with no indication how this compares to MDSC identified in peripheral blood or to those of related myeloid counterparts such as alveolar macrophages and monocytes. Most phenotypic and functional markers have been described in oncological studies but have not yet been validated in TB. Using a panel of 43 genes selected from pathways previously shown to contribute to tumor-derived MDSC, we set out to evaluate if the expression of these additional functional markers and properties may also be relevant to TB-derived MDSC. Differential expression was investigated between MDSC, alveolar macrophages and monocytes enriched from bronchoalveolar lavage fluid and peripheral blood of patients with active TB, patients with other lung diseases (OLD). Results demonstrated that anatomical compartments may drive compartment-specific immunological responses and subsequent MDSC immunosuppressive functions, demonstrated by the observation that MDSC and/or monocytes from PB alone can discriminate, *via* hierarchical clustering, between patients with active TB disease and OLD. Our data show that the gene expression patterns of MDSC in peripheral blood and bronchoalveolar lavage fluid do not cluster according to disease states (TB vs OLD). This suggests that MDSC from TB patients may display similar gene expression profiles to those found for MDSC in cancer, but this needs to be validated in a larger cohort. These are important observations for TB research and may provide direction for future studies aimed at repurposing and validating cancer immunotherapies for use in TB.

## Introduction

Tuberculosis (TB), one of the most prominent communicable diseases in existence, is caused by *Mycobacterium tuberculosis* (M.tb) infection (1). While some people remain asymptomatically infected, others develop active disease. A major aim of TB research efforts is to characterize host immune components contributing to TB disease susceptibility and resistance (2). Host immune modulators are currently explored as a popular approach to enhance immune responses through novel vaccines and drugs thereby reducing susceptibility to infection, delaying disease progression and accelerating cure (2–4).

Myeloid derived suppressor cells (MDSC) are a heterogenous population of immature myeloid cells, with potent immune suppressive functions (5). MDSC are further grouped into subsets, such as immature MDSC progenitor cells known as early-stage MDSC (e-MDSC), polymorphonuclear MDSC (PMN-MDSC) and monocytic MDSC (M-MDSC) (6–8). The most recently described subset of eosinophilic MDSC (Eo-MDSC) expand our understanding of the suppressive granulocyte lineage (9). A multitude of immunosuppressive mechanisms have been reported for tumor-derived MDSC, presumably reflective of the heterogeneity and functional redundancy of this cell population. While these mechanisms are designed to suppress excess inflammation, they are frequently demonstrated to rather inactivate pivotal immune responses required for protection against tumor cells and pathogenic infections alike (10, 11).

As a major immunotherapeutic target in the field of oncology, studies of tumor-derived MDSC suggest that the factors involved in their differentiation, expansion and activation can be characterized in two classes. Firstly, factors stimulating myelopoiesis and inhibiting differentiation from immature myeloid cells (IMC) into mature myeloid cells (MMC) (5, 12, 13) enhance the accumulation of IMC in lymphoid organs and the tumor microenvironment (cancer). Such factors include interleukin (IL)-6 and vascular endothelial growth factor alpha (VEGFA) which activate the signal transducer and activator of transcription factor 3 (STAT3) signaling pathway (14–17). Secondly, factors such as such as the transcription factors c-myc and C/EBP homologous protein (CHOP), reactive oxygen species (ROS), inducible nitric oxide synthases (iNOS), arginase, IFN-y, TNF-α, IL-13, S100A8/9, transforming growth factor beta (TGF-β), IL-10, and prostaglandin E2 (PGE2) are involved in the pathological expansion and activation of hematopoietic stem cells (HPC) into IMC and eventually MDSC (5, 16). In cancer, these factors are secreted by activated T cells and tumor stromal cells (5, 14–16).

Suppressive myeloid cells have long been known to be expanded in cancer (18, 19), but the identification of their expansion in other inflammatory conditions including infectious diseases such as active TB disease, are more juvenile. Increased MDSC frequencies have been measured in the peripheral blood of patients with active pulmonary TB disease (20–22), however, little is known about the true phenotype and immunosuppressive mechanisms of these cells in TB. Importantly, the frequency and signaling pathways of MDSC at the site of TB disease (the lung) is also unknown with no indication how this compares to those identified in the blood or to those of related myeloid counterparts such as alveolar macrophages, monocytes, dendritic cells, or neutrophils. In recent studies it was shown that MDSC exhibit monocytic and granulocytic morphologies, although M-MDSC appear more relevant in the context of M.tb-infected participation, due to its phagocytic potentials (20, 23, 24).

In the field of immuno-oncology, the minimally accepted set of surface markers for phenotypic classification of human MDSC is HLA-DR, CD33, CD11b and CD14 (M-MDSC) or CD15 (PMN-MDSC) (25). These markers have also been adopted in the human TB MDSC research field, specifically to characterize blood-derived MDSC. Many additional phenotypic and functional markers have, however, recently been described in oncological studies, relating to the function and ontology of MDSC, the most promising being LOX-1 (PMN-MDSC) and S100A9 (M-MDSC) (26–28). Here we set out to evaluate if the expression of these and other functional markers and functional properties associated with MDSC in cancer, may also be relevant to TB-derived MDSC. Additionally, we hypothesize that MDSC from the blood of TB patients could display similar characteristics to lung-derived MDSC but would differ to those of other control myeloid subsets. We make use of myeloid cells from peripheral blood cells and bronchoalveolar lavage cells from of individuals with active TB or with non-TB MDSC-inducing lung diseases, other than TB, to assess expression of genes previously shown to be linked to MDSC in lung malignancies. Specifically, we compare expression between MDSC, alveolar macrophages (AM) and monocytes, to determine how these compare between myeloid subsets.

## Materials and Methods

### Ethics Approval and Statement

Ethical approval was obtained from the Health Research Ethics Committee of Stellenbosch University as part of ongoing clinical studies, namely TANDEM: Concurrent Tuberculosis and Diabetes Mellitus - Unravelling the causal link and improving care (N13/05/064); ICIDR: Biology and Biosignatures of Anti-Tuberculosis Treatment Response (NIH/U01/AI115619); Screen TB study: Evaluation of host biomarker-based point-of-care tests for targeted screening for active TB (N16/05/070 and N16/04/050); The effect of cigarette smoking on host and M.tb responses (N10/08/276). The study was conducted in accordance with the Declaration of Helsinki and International Conference on Harmonisation guidelines.

### Participant Recruitment and Sample Collection

**Figure 1:**
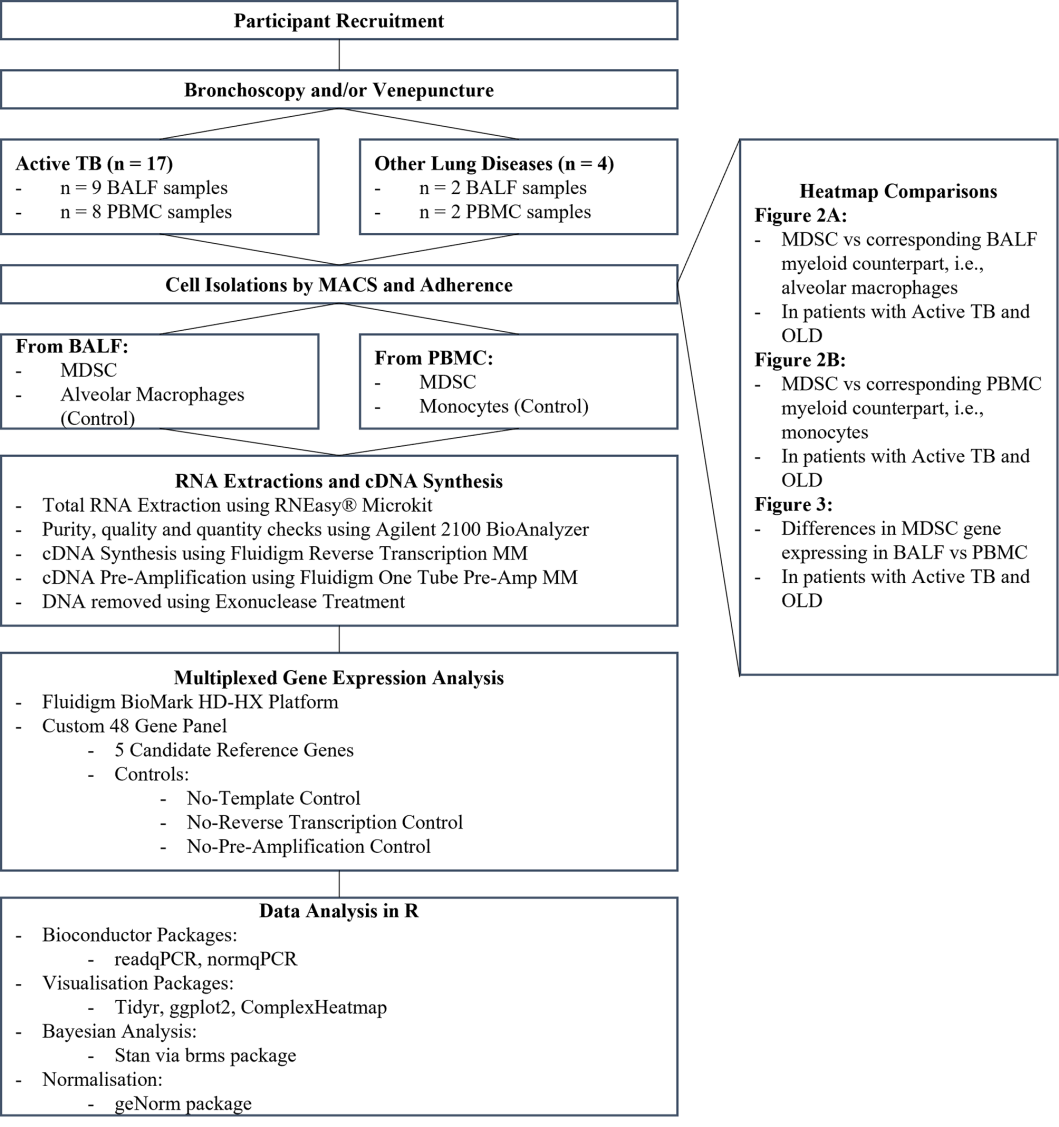
Experimental design and workflow.

Participants were enrolled from a sub-district in Cape Town, South Africa and written informed consent was obtained. Participants were grouped as healthy household contacts (HHC), other lung diseases (OLD) or active TB (**Figure 1**). The criteria for inclusion in the HHC or OLD groups were no clinical, radiological, or microbiological signs of active TB with a negative sputum GeneXpert. The inclusion criteria for active TB were clinical signs of TB (symptoms such as, persistent cough, fever, night sweats, weight loss, loss of appetite) with a chest x-ray screen showing signs of TB, or a positive sputum culture test (culture negative TB included if positive for GeneXpert). Participants were excluded if they had a history of previous TB, tested positive for diabetes, or tested positive for HIV. Peripheral blood (PB) was obtained by venepuncture into NaHep Vacutainers^®^. broncho-alveolar lavage fluid (BALF) was collected by bronchoscopy conducted by an experienced pulmonologist. Samples were processed within 2 hours of collection.

### Immune Cell Enrichment

Peripheral blood mononuclear cells (PBMC) were isolated from PB using the standard ficoll density medium separation method (GE Healthcare, Piscataway, New Jersey, United States of America). BALF was filtered using a 70 µm cell strainer and the cell pellet was washed using RPMI supplemented with 5% FBS. MDSC were enriched from PBMC and BALF cells using magnetic-activated cell sorting (MACS) through a series of negative and positive selection steps, to obtain the CD3^-^/HLA-DR^-^/CD33^+^ cell fraction enriched for MDSC (MiltenyiBiotec, Germany). In parallel, for each participant, monocytes (from PBMC) and AM (from BAL) were enriched from the HLA-DR^+^ fraction through plastic adherence in an overnight culture. Cells were counted and viability assessed through Trypan Blue staining. Samples displayed viability >95% and the quality and efficacy of the isolation was assessed by flow cytometric assessment of peripheral monocytes (CD33^+^HLA-DR^+^-PE) and MDSC (HLA-DR^-^; CD33^+^ BV510) (BD FACS Canto II). Data was analysed using FlowJo (version 10). Enriched fractions were stored in RLT Plus^®^ Buffer (Qiagen, Germany) at -80°C for batch analysis.

### RNA Extraction, cDNA Synthesis and Quantitative Real-Time PCR (qRT-PCR)

Total RNA was isolated from enriched cell fractions using the RNEasy^®^ Micro Kit (Qiagen, Germany) according to the manufacturer’s instructions and stored at -80°C. The RNA purity, quality and quantity were assessed using the Agilent 2100 BioAnalyzer (Agilent Technologies Inc.) An RNA integrity number (RIN) score above 7 was considered sufficient for downstream processing. Isolated RNA was thawed on ice and cDNA synthesized using the Fluidigm Reverse Transcription Master Mix (Fluidigm PN 100-6297) and a conventional thermal cycler (Life Technologies, United States of America). Aliquots of the cDNA were further subjected to either 16 or 20 pre-amplification cycles using the Fluidigm One Tube PreAmp Master Mix (Fluidigm PN 100-5580) and DNA was removed by exonuclease-I treatment (PN M0293S/L; 20 U/µl).

### Multiplexed Gene Expression Analysis

A customised 48-gene panel (Delta Gene Assay, Fluidigm, California, USA) was designed, based on phenotypic and functional markers related to MDSC as reported in the literature from *in vitro* or *in vivo* studies of various diseases in humans or animal models (**Table 1**). Included in these were 5 candidate reference genes: ACTB (NM_001101.N), G6PD (NM_000402.N), GAPDH (NM_001289745), RPS12 (NM_001016.N), and RPS18 (NM_022551.N). The multiplex qRT-PCR was conducted according to manufacturer’s instructions (Fluidigm BioMark HD-HX platform, California, United States of America). All genes were assayed in duplicate.

**Table 1:** List of genes analysed.

Gene	Description	Gene	Description
ACTB^*^	Actin beta	IL6	Interleukin 6
G6PD^*^	Glucose-6-phosphate dehydrogenase	ITGAM	Integrin subunit alpha M
GAPDH^*^	Glyceraldehyde-3-phosphate dehydrogenase	ITGAX	Integrin subunit alpha X
RPS12^*^	Ribosomal protein S12	MAPK14	Mitogen-activated protein kinase 14
RPS18^*^	Ribosomal protein S18	MAPK3	Mitogen-activated protein kinase 3
ARG1	Arginase 1	MIR146A	MicroRNA 146a
CAV1	Caveolin 1	MIR17	MicroRNA 17
CD14	Cluster of Differentiation 14	MIR223	MicroRNA 223
CD274 (PDL1)	Cluster of Differentiation 274	MIR494	MicroRNA 494
CD33	Cluster of Differentiation 33	MRC1	Mannose receptor, C type 1
CD36	Cluster of Differentiation 36	MTOR	Mechanistic target of rapamycin
CPT1A	Carnitine palmitoyltransferase 1A	NOS2	Nitric oxide synthase 2
CSF2RA	Colony stimulating factor 2 receptor alpha subunit	OLR1	Oxidized low density lipoprotein receptor 1
FASLG	Fas ligand	PLIN1	Perilipin 1
FUT4	Fucosyltransferase 4	PTGS2	Prostaglandin-endoperoxide synthase 2
HADHA	Hydroxyacyl-CoA dehydrogenase	S100A8	S100 calcium binding protein A8
HLA-DRA	Major histocompatibility complex class II, D-related protein alpha	SIRPA	Signal regulatory protein alpha
HMGB1	High mobility group box 1	SLC27A4	Solute carrier family 27 member 4
IDO1	Indoleamine 2,3-dioxygenase 1	STAT1	Signal transducer and activator of transcription 1
IFNG	Interferon gamma	STAT3	Signal transducer and activator of transcription 3
IL10	Interleukin 10	STAT6	Signal transducer and activator of transcription 6
IL17A	Interleukin 17A	TGFB1	Transforming growth factor beta 1
IL1B	Interleukin 1 beta	TNF	Tumor necrosis factor
IL4	Interleukin 4	VEGFA	Vascular endothelial growth factor A

*Reference genes.

Controls included no-template controls, no-reverse-transcription controls, and no-pre-amplification controls. Upon successful completion of the Fluidigm assay the Ct-values (the fractional number of cycles required for the signal to cross the detection threshold), proportional to the inverse gene expression levels, were exported for further analysis.

### Data Processing and Statistical Analysis

Data processing and analysis was performed using R [“R Core Team (29)] along with the BioConductor packages readqPCR and normqPCR. R packages tidyr, ggplot2 and ComplexHeatmap were used for processing and visualization of the data. Bayesian statistical analysis was done using Stan **(** 30 **)** *via* the brms package **(** 31 **)**. Analysis of differences in expression levels (Ct-values) was done using Bayesian multilevel models with either cell-type or sample-type as the fixed-effect modelled using a student t-distribution and subject and pre-amplification cycle as random intercept effects with a default flat prior on the effect estimate. Sampling was set at 5000 iterations, including a warmup of 1500, using 4 chains and an adapt_delta value of 0.9. Correlation analysis was done using the Spearman rank method.

## Results

### Participant Characteristics, Sample Size and Immune Cell Enrichment

Peripheral blood (n=8) and BALF (n=9) samples were collected from participants upon diagnosis of active pulmonary TB disease prior to initiation of standard antibiotic treatment and controls with lung disease other than TB, residing in the same community (n=4) (**Table 2**). Of the OLD patients, the OLD included pulmonary complications associated with leukaemia, a pulmonary fungal infection, lung cancer, and sarcoidosis.

**Table 2:** Sample set selection.

Group	BALF cells(MDSC and AM)	PBMC(MDSC and monocyte)
Active TB	7 (Paired MDSC and AM)1 (MDSC), 1 (AM)	8 (Paired MDSC and Monocyte)
Other Lung Diseases	2	2

MDSC (CD3^-^/HLA-DR^-^/CD33^+^) were enriched from PBMC and BALF, while AMs and monocytes (CD3^-^/HLA-DR^+^/CD33^+^) were enriched from BALF and PBMC, respectively, using MACS isolation technology, as compartmental control populations. Purity checks were run on the enriched MDSC and monocyte populations by flow cytometry and were demonstrated to have an average purity of 73% and 100%, respectively. Because of the lower purity of the MDSC population, this is a population enriched for MDSC, rather than a pure population. Purity checks could not be performed on the AMs owing to the known issues with autofluorescence in these cells because of the high level of carbon loading within the macrophage population of participants from the Western Cape province of South Africa (32). Literature shows that between 85-96% of cells from the BALF of the human lung are alveolar macrophages (33 **,** 34). Therefore, while not 100% pure, the cells obtained from the lung in this study are enriched for AMs.

### Reference Gene Selection

To compare gene expression levels between cell or sample types, the absolute expression levels were first normalized using the Ct values for a set of reference genes. A panel of five candidate reference genes was selected as endogenous controls based on the criterion of constitutive expression, regardless of cell, tissue, or sample type. These were: ACTB, G6PD, GAPDH, RPS12 and RPS18. We selected an optimal subset of these candidate reference genes based on an assessment of gene expression stability as determined by the method of Vandersmpele et al. using the geNorm package (35). We found good average stability (M-values, higher values denote lower stability) of the reference sets, improving with the progressive exclusion each least stable gene. The pairwise ratios of the M-values showed nominal changes in stability with each additional reference gene, although we observed a notable increase with the addition of a fifth gene in some sample-types. The implication being that there is little benefit to be gained from using more than the recommended three reference genes. As ACTB and RPS18 were the least stable references in the 16- and 20-times pre-amplification assays respectively, we chose to exclude both. Normalised delta-Ct values were therefore calculated based on the geometric mean expression of the G6PD, GAPDH and RPS12 genes.

### Hierarchical Clustering and Statistical Analysis of Normalized Expression Data

#### Gene Expression Profiles of MDSC and/or Monocytes in the Blood Discriminate Between Patients With TB Versus Those With OLD

**Figure 2:**
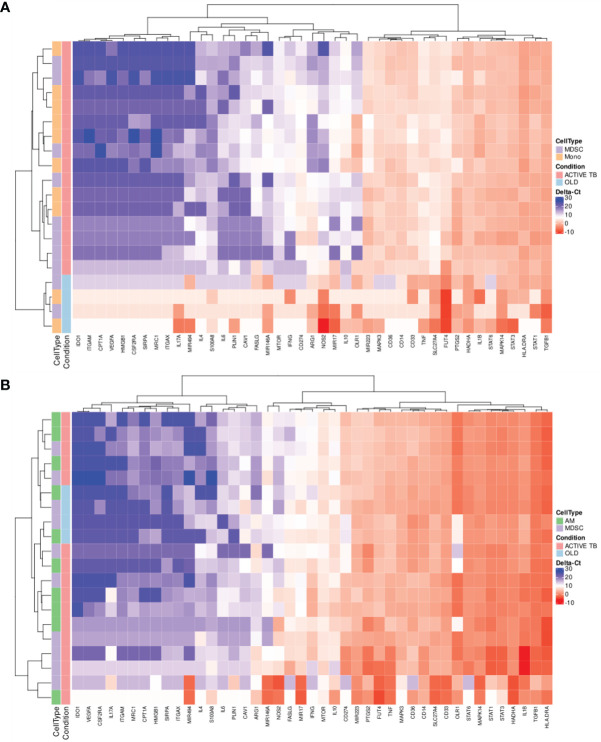
Scaled gene expression patterns of **(A)** MDSC and monocytes in PB (16x pre-amplification), and **(B)** MDSC and AM in BALF (16x pre-amplification). Lower expression (higher ΔCt-values) is indicated by blue. Hierarchical clustering of similar expression patterns across subject and genes is indicated by the dendrograms.

It remains unclear how the phenotype and function of MDSC differ in various chronic inflammatory conditions. This information will be critical in selecting and designing targeted host-directed therapeutic (HDT) strategies and inform repurposing of compounds being tested in immune-oncology. We measured the expression of 43 target genes, previously shown to have relevance in MDSC phenotype and function in immune-oncology, in peripheral blood-derived MDSC and monocytes from TB patients and those with OLD, (**Figure 2A**). We observed no obvious distinction between the expression patterns of either peripheral MDSC or monocytes between patients with TB versus those with OLD. We also investigated changes in gene expression of BAL-derived MDSC and monocytes between patients with active TB and OLD. Hierarchical cluster analysis showed no obvious distinction between the expression patterns of either BAL-derived MDSC or AM between TB and OLD (**Figure 2B**).

Gene expression can also be employed for the discovery of discriminatory gene or multigene signatures in disease. Interestingly, hierarchical cluster analysis of gene expression levels in both MDSC and monocytes in PB displayed clear differences between patients with active TB and those with OLD, resulting in complete separation of the two conditions (**Figure 2A**).

#### Blood-Derived MDSC and Monocytes From TB Patients Display Differential Enrichment in Selected Gene Transcriptional Levels

Hypotheses as to the source of circulating MDSC in TB patients include the export of immature cells into the blood stream through emergency myelopoiesis or the reprogramming of exported peripheral monocytes (18, 36, 37). Some have shown that these reprogrammed monocytes also have the same phenotype and function as M-MDSC in cancer patients (26). We therefore assessed the transcriptional differences of the 43 genes, previously shown to demonstrate differential abundance between monocytes and MDSC in cancer, in TB patient-derived MDSC and TB patient-derived monocytes. Statistical analysis of gene expression between MDSC and monocytes in PB of active TB patients yielded differences in ΔCt-values in 12 genes at the 95% Bayesian highest posterior density interval (HDI) level (**Table 3**, starred values). A further 9 genes (**Table 3**, not starred) showed possible differential expression at the 90% HDI level and require further investigation. Of these 21 genes, seven showed a ΔΔCt of at least 2, equivalent to a difference in expression of a factor of at least 4. Of these, *ARG1, CPT1, ITGAM, IL4* and *IL17A* showed higher expression in MDSC than in monocytes, whereas *IFNG* and *FASLG* expression was lower in MDSC. The changes in all 43 genes’ ΔCt-values can be found in **Supplementary Figure 1**.

**Table 3:** Differential gene expression levels between MDSC and Monocytes from PB, with respect to expression levels in Monocytes.

Gene	Median ΔΔCt	MAD	Q2.5	Q5	Q95	Q97.5	Expression Factor
*ARG1*	-2.113^*^	0.627	-3.463	-3.231	-1.086	-0.866	↑ 4.3
*CD33*	-0.804	0.394	-1.613	-1.460	-0.106	0.041	↑ 1.7
*CD36*	-0.600	0.293	-1.187	-1.085	-0.104	0.002	↑ 1.5
*CPT1A*	-3.137^*^	0.751	-4.632	-4.375	-1.804	-1.532	↑ 8.8
*CSF2RA*	-2.048^*^	0.874	-3.798	-3.483	-0.502	-0.198	↑ 4.1
*FASLG*	3.164	1.845	-0.549	0.089	6.236	6.924	↓ 9.0
*FUT4*	-0.651^*^	0.316	-1.624	-1.380	-0.189	-0.103	↑ 1.6
*IDO1*	-1.635	0.865	-3.459	-3.150	-0.149	0.190	↑ 3.1
*IFNG*	4.446^*^	1.734	0.930	1.586	7.452	8.076	↓ 21.8
*IL17A*	-3.439	1.804	-7.067	-6.455	-0.407	0.169	↑ 10.8
*IL4*	-3.038	1.641	-6.377	-5.808	-0.276	0.346	↑ 8.2
*ITGAM*	-2.795^*^	0.532	-4.013	-3.789	-1.926	-1.723	↑ 6.9
*ITGAX*	-2.353^*^	1.017	-4.392	-4.044	-0.623	-0.263	↑ 5.1
*MAPK14*	-0.366	0.199	-0.841	-0.740	-0.001	0.068	↑ 1.3
*MIR223*	-1.025^*^	0.379	-1.831	-1.685	-0.402	-0.263	↑ 2.0
*NOS2*	-3.014	1.484	-6.065	-5.547	-0.483	0.032	↑ 8.1
*PTGS2*	-0.747^*^	0.353	-1.415	-1.303	-0.181	-0.064	↑ 1.7
*S100A8*	-1.491^*^	0.529	-2.556	-2.384	-0.605	-0.417	↑ 2.8
*SIRPA*	-2.301^*^	0.872	-4.016	-3.740	-0.799	-0.470	↑ 4.9
*SLC27A4*	0.59	0.324	-0.058	0.056	1.150	1.263	↓ 0.7
*VEGFA*	-1.901^*^	0.845	-3.622	-3.308	-0.478	-0.157	↑ 3.7

Only results where the 90% HDI of the ΔΔCt value excluded 0 are shown. ΔΔCt is with respect to expression levels in Monocytes. * Indicates results where the 95% HDI of the Ct value excluded 0. MAD, Median Absolute Deviation.

#### Lung-Derived MDSC and Macrophages From TB Patients Display Differential Enrichment in Selected Gene Transcriptional Levels

The mechanism(s) employed by MDSC to suppress T-cell responses in TB is yet unknown and investigation into these will improve selection of precision strategies to specifically target MDSC. We included in our gene panel a selection of genes known to mediate the suppressive function of tumor-derived MDSC, to evaluate if these are enriched also in TB-derived MDSC. Importantly, the suppressive function of MDSC is likely to feature at the inflammation site, which in cancer is at the tumor site, or in the case of TB, the lung infection site. MDSC and AM, isolated from lung-derived BALF of TB patients, yielded gene expression differences in ΔCt-values in 6 genes at the 95% HDI level (**Table 4**, starred values). Four of these: *CAV1*, *IL10*, *IL6* and *MIR146A*, showed a difference in ΔCt of at least 4. In all four, expression was lower in MDSC than in AM. Only *IL1B* expression was higher in MDSC versus AM. The changes in all 43 genes’ ΔCt-values can be found in **Supplementary Figure 2**.

**Table 4:** Differential gene expression levels between MDSC and AM in BALF, with respect to expression levels in AM.

Gene	Median ΔΔCt	MAD	Q2.5	Q5	Q95	Q97.5	Expression Factor
*CAV1*	2.756^*^	1.199	0.587	0.926	4.720	5.081	↓ 6.8
*CD36*	1.089^*^	0.409	0.300	0.424	1.832	2.006	↓ 2.1
*IL10*	2.451^*^	1.151	0.649	0.912	4.785	5.266	↓ 5.5
*IL1B*	-0.873^*^	0.364	-1.628	-1.484	-0.207	-0.050	↑ 1.8
*IL6*	3.528^*^	0.999	1.437	1.761	5.146	5.472	↓ 11.5
*MIR146A*	3.339^*^	1.364	0.548	1.028	5.632	6.114	↓ 10.1

Only results where the 90% HDI of the ΔΔCt estimate excluded 0 are shown. ΔΔCt is with respect to expression levels in AM. * Indicates results where the 95% HDI of the Ct value excluded 0. MAD, Median Absolute Deviation.

#### Gene Expression Profiling Suggests That MDSC Phenotype and Function Differ Depending on Anatomical Compartments in TB Patients

**Figure 3:**
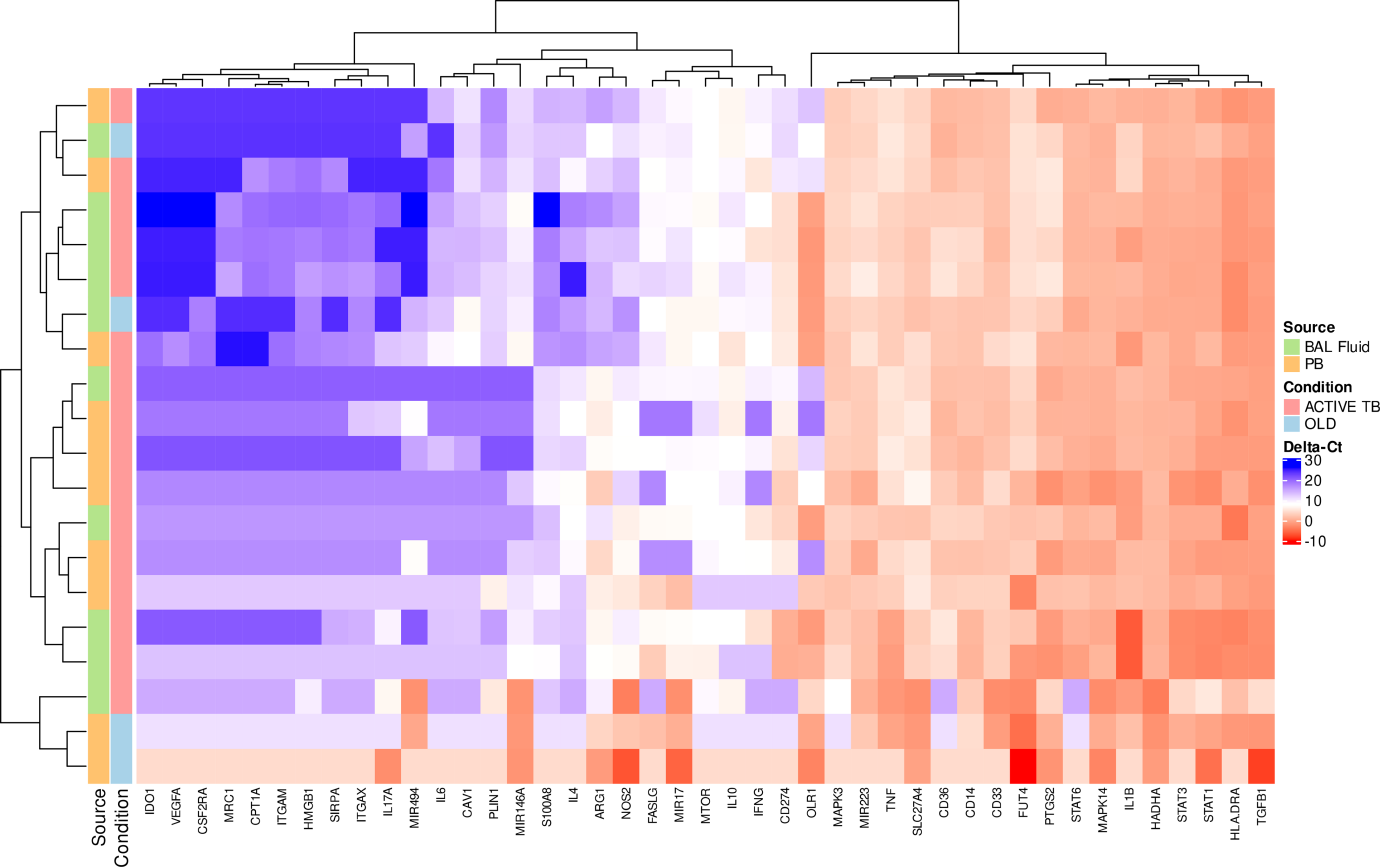
Scaled gene expression patterns of MDSC in BALF and PB (16x pre-amplification). Lower expression (higher ΔCt-values) is indicated by blue. Hierarchical clustering of similar expression patterns across subject and genes is indicated by the dendrograms.

The fate of circulating MDSC in pulmonary TB patients has not been determined, but the theory is that MDSC released from the bone marrow migrate *via* the blood stream to the site of infection in the lung. How the transcriptional profile of circulating MDSC compares to MDSC entering the disease site, remains to be determined. Statistical analysis of gene expression between MDSC in PB and BALF of active TB patients yielded differences in ΔCt-values in 7 genes at the 95% HDI level (**Table 5**, starred values). Five of these: *IFNG*, *IL1B*, *OLR1*, *SLC27A4* and *TNF*, showed a difference in ΔCt of at least 2 in expression. In all five, expression was lower in PB MDSC than in BALF MDSC. Of note is *OLR1* which was lower by a factor of 180.9 in MDSC in PB compared to BALF. A further 3 genes showed possible differential expression at the 90% HDI level (**Table 5**, not starred). Hierarchical cluster analysis of gene expression levels in MDSC in PB and BALF did not discriminate patients with active TB from those with OLD (**Figure 3**). We also observed no obvious distinction between the expression patterns of the two cell-types. As PB is a much more accessible sample-type that BALF, we did a correlation analysis of the gene expression levels between the two compartments. The results showed moderate to no correlation (**Supplementary Table 1**). The changes in all 43 genes’ ΔCt-values can be found in **Supplementary Figure 3**.

**Table 5:** Differential gene expression levels between MDSC in BALF and PB, with respect to expression levels in BALF.

Gene	Median ΔΔCt	MAD	Q2.5	Q5	Q95	Q97.5	Expression Factor
*CD36*	-1.925^*^	0.726	-4.563	-3.857	-0.739	-0.470	↑ 3.8
*FASLG*	4.324	2.226	-0.326	0.503	8.153	8.941	↓ 20.0
*HADHA*	0.996	0.635	-0.206	0.034	2.094	2.211	↓ 2.0
*IFNG*	4.656^*^	2.501	0.101	0.942	8.561	9.416	↓ 25.2
*IL1B*	2.146^*^	1.106	0.239	0.648	4.150	4.507	↓ 4.4
*MRC1*	3.001	1.691	-0.396	0.226	5.800	6.411	↓ 8.0
*MTOR*	1.279^*^	0.586	0.104	0.306	2.277	2.508	↓ 2.4
*OLR1*	7.499^*^	2.272	2.783	3.603	11.422	12.352	↓ 180.9
*SLC27A4*	2.412^*^	0.821	0.763	1.038	3.884	4.211	↓ 5.3
*TNF*	2.604^*^	0.910	0.632	1.037	4.195	4.548	↓ 6.1

Only results where the 90% HDI of the ΔΔCt estimate excluded 0 are shown. ΔΔCt is with respect to expression levels in BALF. * Indicates results where the 95% HDI of the Ct value excluded 0. MAD, Median Absolute Deviation.

## Discussion

The phenotype and function of MDSC in the context of TB remains incompletely defined. Most importantly, PB blood has long been used for measurement of soluble mediators and immune cell profile during TB disease, as proxy for the immunological profile at the site-of-disease **(** 38). Previous studies have focussed on profiling actively expressed receptors on the surface of MDSC in the periphery during active TB and their soluble mediators, but few have investigated the gene expression profiles of MDSC isolated from different immunological compartments **(** 20, 24, 39, 40). The immune phenotype of MDSC in relation to other related immune cell types have also not been compared between the periphery and the lungs during active TB disease. To address these shortfalls, we performed a pilot, observational study in which we assembled a panel of 43 genes from pathways previously shown to contribute to MDSC phenotype, activation, and function in tumor biology. Since MDSC are actively targeted in immune-oncology, owing to the comprehensive knowledge available on the role of these cells in failed host immunity to cancer cells (41), we aimed to identify which MDSC-related pathways also applies to TB. We measured the expression of these genes in MDSC, AM and monocytes from BALF and PB of patients with TB, and those of patients with lung disease other than TB, previously described to show an upregulation in MDSC. This information is important to inform the selection of HDT strategies in TB, specifically those that may be repurposed from the immune-oncology field should the gene expression profiles of MDSC in TB and cancer (OLD) be similar.

In this study, we measured the expression of 43 target genes previously shown to have relevance in MDSC phenotype and function in immune-oncology, to determine their relevance in TB-derived MDSC. We compared these MDSC expression signatures to those observed in control monocyte and AM populations owing to the majority of MDSC in the blood being of the monocyte lineage. Studies have demonstrated that M-MDSC are the predominant subset in the periphery (20, 21, 24), and data from immune-oncology studies have demonstrated that M-MDSC entering the tumor are capable of differentiating into tumor-associated macrophages (TAMs), supporting the use of alveolar macrophages as a control population from the lung **(** 42).

In this study we did not observe any significant differences in gene expression in blood-derived MDSC between active TB patients and those with OLD. We also did not measure any significant changes in gene expression of BAL-derived MDSC between active TB patients and those with OLD. The same was also observed for blood- and BAL-derived monocytes between TB patients and OLD. Although there were only two OLD patients in the dataset, we observed a clustering capable of distinguishing between the two groups of patients using PB. This was true for both MDSC and monocytes which suggests that it may be worthwhile investigating the possibility of a PB, cell type-specific RNA diagnostic signature for TB in a larger study. Interestingly, no such discriminating pattern was observed for either MDSC or AM isolated from BALF. These results suggest that there may still be value in investigating PB-specific RNA biomarkers, specifically for the discrimination of OLD from TB disease, and that perhaps previous studies looking at secreted soluble mediators should rather investigate at a gene expression level.

Monocytes and MDSC derived from the PB of TB patients recruited in this study demonstrated 21 differentially expressed genes at both the 90% and 95% Bayesian HDI level. It is interesting to note that of the 21 differentially expressed genes between MDSC and monocytes in PB, many of the genes were associated with the PMN-MDSC subset specifically. These included *FUT4*, *IFNG*, *ITGAM*, *MIR223*, *PTGS2*, *VEGFA* and *MAPK14* to name a few. Conventional studies using PB rely on the cryopreservation of PBMC for batched downstream analyses which is known to result in the loss of the majority, if not the entire, PMN-MDSC subset (43). Because this study made use of PBMC immediately stored in an RNA-preserving buffer, we were able to preserve PMN-MDSC-specific RNA for differential expression analyses. It is clear from these results that the PMN-MDSC subset is severely under-reported in the context of active TB disease, and its functional role has subsequently not been accounted for. It would be prudent for future studies to identify realistic PMN-MDSC proportions in the PB of patients with active TB disease and focus on their immunosuppressive role.

Among the differentially expressed genes were also *IFNG* and *IL17A*, which were 21.8 times lower and 10.8 times higher in MDSC than in monocytes, respectively. The former finding was not unexpected. Current knowledge suggests that, during active TB disease, *ARG1* and *NOS2* play a pivotal role in the suppression of T cell proliferation and IFN-γ production, thereby preventing the efficient killing of intracellular pathogens in macrophages and impairing T cell activation (21, 44–46). The 10-fold upregulation of *IL17A* alludes to the role of IL-17-producing CD4^+^ T cells (Th17 cells) during tumor development, which drive tumorigenesis (47). In contrast, IL-17A is known to be protective against mycobacterial infection in the host, and a lack thereof increases TB susceptibility (48). Given that the main producing cell type of IL-17A remains CD4^+^ and CD8^+^ T cells, evidence suggests that TAMs (derived from M-MDSC) are capable of producing IL-17A and may therefore be present in the PB of active TB patients (49). Other differentially expressed genes of note were *FASLG* (9 times lower in MDSC than monocytes), and *CPT1A* (8.8 times higher in MDSC than monocytes). The expression of *FASLG* on the surface of MDSC is responsible for inducing the apoptosis of T cells by binding with T cell-expressed Fas (50, 51). Data from *FASLG* deficient mice demonstrate that the lack thereof results in a significant reduction of MDSC in the tumor microenvironment, but more so of the PMN-MDSC subset, suggesting that the lack of FASLG skews the remaining MDSC population to the M-MDSC, and therefore more immunosuppressive, phenotype (52). Considering that this 9 times reduction in MDSC compared to monocytes occurred within the PB suggests that circulating MDSC lack the FasL-Fas immunosuppressive mechanism, which was supported by our data in **Table 5**. This demonstrated that the expression of *FASLG* on MDSC was 20 times lower in PB compared to BALF. *CPT1A* is a mitochondrial enzyme involved in fatty acid metabolism, the lipid metabolic state preferred by MDSC to which their immunosuppressive potentials can be attributed (53, 54). The upregulation of this gene was not unexpected as it is in line with immune-oncology findings, but it is promising that these findings are similar between cancer and TB.

In contrast to the abundance of differentially expressed genes between MDSC and monocytes from PB, we identified only 6 genes that were differentially expressed between MDSC and AM isolated from the BALF of active TB patients. These included *CAV1*, *CD36*, *IL10*, *IL1B*, *IL6*, and *MIR146A*. Physiologically, the suppressive function of MDSC is likely to feature most notably at the inflammation site, which in cancer is at the tumor site, or in the case of TB, the lung. We have demonstrated that lung-derived MDSC and macrophages from active TB patients do indeed display differential enrichment in selected gene transcripts, which could inform the immunosuppressive mechanisms of lung-derived MDSC during active TB disease. IL-6, IL-10 and IL1-β are well known cytokines involved in the induction/expansion of MDSC, with IL-10 also playing a role in their effector functions at the site of disease (44, 55–58). Of interest is the observed downregulation of *CAV1* and *CD36*, both of which are involved in lipid metabolism and metabolic reprogramming of MDSC, and mycobacterial infections, and are associated with poor clinical outcomes in malignant tumors (59, 60). Previous mycobacterium-specific research demonstrated that vesicular TLR2/Cav-1 signalling, while dispensable for *Mycobacterium bovis* internalization specifically, is required for T cell suppressive functions within the MDSC milieu (61). Five out of the six genes were less differentially expressed in the MDSC population compared to the AM, suggesting that AM are responsible for the expression of these genes, including IL-10, possibly to induce the expansion of MDSC at the site of disease, while utilising CAV1/CD36-driven lipid metabolism pathways to create a niche for mycobacterial survival (62, 63), thereby exacerbating disease progression.

The fate of circulating MDSC in active TB patients has not been determined, but the theory is that MDSC released from the bone marrow migrate *via* the blood stream to the site of infection in the lung. How the transcriptional profile of circulating MDSC compares to MDSC entering the disease site, therefore remains to be determined and is of importance to future HDT strategies. Our comparison of gene expression levels in MDSC from PB *vs.* BALF showed that, at least for the 10 genes listed in **Table 5**, the gene expression pattern observed in PB does not reflect levels seen in the lung. This was confirmed by the observation that there was little to no correlation between the expression levels in MDSC from the two sites for all genes (**Supplementary Table 1**), as well as the high expression factor differences between the two compartments. It is clear from this data that there may be some phenotype and functional profile differences depending on the anatomical compartment from which MDSC were derived. Unfortunately, though, hierarchical cluster analysis demonstrated that, at least during this study, the gene expression profiles of MDSC in PB compared to BALF could not discriminate patients with active TB from those with OLD. Of note from this comparison is the observation of *OLR1* being lower by a factor of 180.9 in MDSC in PB compared to BALF. *OLR1* codes for the lectin-type oxidized low-density lipoprotein receptor 1 (also known as LOX-1), and is highly expressed in PMN-MDSC (27). With this in mind, previous studies suggesting M-MDSC are the dominant subset of MDSC within the PB compartment are supported, further suggesting the PMN-MDSC subset may dominate the lung compartment, but this needs to be validated.

A significant limitation of this study is that it was unfortunately not possible to obtain paired BALF and PB samples from the same patients which would have enhanced the power of this study. Working with clinical samples is known to be challenging in the TB field; this is especially true for the collection of BALF samples as the bronchoscopy procedure is more invasive and uncomfortable than venepuncture. Often, paired blood specimens cannot be collected in tandem, and additionally, it is likely that the inability to discriminate gene expression profiles of MDSC between other cells or anatomical compartments, is as a result of the small sample size available during this study. A follow-up study is currently being conducted by our research group making use of paired samples from a larger number of participants to validate the findings observed during this study. In addition, our sample size for the comparative OLD group was incredibly small and each individual presented with a distinct diagnosis. This is problematic for representative sampling and should be considered when interpreting the results obtained during this study. Future studies may do well to focus on obtaining patient samples specifically from those with lung cancer to draw stronger parallels between the TB and OLD group. Lastly, this study would benefit from additional validation studies with a particular need being the use of a larger cohort with paired samples.

The results achieved from this study support the hypothesis that anatomical compartments may drive compartment-specific immunological responses, which have considerable effects on the MDSC population and their immunosuppressive functions specifically. The most interesting findings are that the gene expression profiles of MDSC and/or monocytes from PB are able to discriminate between patients with active TB disease and OLD, and that differential gene expression in MDSC vs monocytes is highly variable compared to differential gene expression in MDSC vs AM, with the lung compartment demonstrating a preferential trend to upregulate genes involved in MDSC expansion and lipid metabolism. Considering that the gene expression profiles of MDSC in PB and BALF are not capable of clustering according to disease groups (TB or OLD), it is feasible to speculate that MDSC from TB share similar gene expression profiles with those from cancer but needs to be validated in a larger study. These are important observations for the TB field, possibly lending support to repurpose and validate cancer immunotherapies as HDT for active TB disease. This study provides a backbone upon which future studies into MDSC-related gene expression during active TB disease should be based, as a guide for both improvement and targeted investigations.

## Data Availability Statement

The data presented in the study are deposited in the GEO repository, accession number GSE197113 (https://www.ncbi.nlm.nih.gov/geo/query/acc.cgi?acc=GSE197113).

## Ethics Statement

The studies involving human participants were reviewed and approved by Health Research Ethics Committee of Stellenbosch University. The patients/participants provided their written informed consent to participate in this study.

## Author Contributions

This study was conceived by NP and designed with support from GS and GW. BL, MM, and ME conducted the experiments and analysed the data with support from AP-N, GS, and TS. LK, GS, and NP wrote the manuscript with comments from GW. SA and STM were responsible for participant recruitment, while BA and CK were responsible for the bronchoscopies performed to acquire the BAL fluid. All authors contributed to the article and approved the submitted version.

## Funding

This work was supported by the National Institute of Health (NIH) International Collaborations in Infectious Disease Research (ICIDR): Biology and Biosignatures of anti-TB Treatment Response (U01 AI115619), European & Developing Countries Clinical Trials Partnership (EDCTP; CDF1546), and South African Research Chair Initiative (SARChI): Biomarkers for TB (SANRF86535).

## Conflict of Interest

The authors declare that the research was conducted in the absence of any commercial or financial relationships that could be construed as a potential conflict of interest.

## Publisher’s Note

All claims expressed in this article are solely those of the authors and do not necessarily represent those of their affiliated organizations, or those of the publisher, the editors and the reviewers. Any product that may be evaluated in this article, or claim that may be made by its manufacturer, is not guaranteed or endorsed by the publisher.

## Supplementary Material

Supplementary Figure 1 ΔCt-values for all 43-genes assessed for differential expression between MDSC and monocytes in the PB of active TB patients. Results are given for the 16X (pink) and 20X (blue) pre-amplification cycles.

Supplementary Figure 2 ΔCt-values for all 43-genes assessed for differential expression between MDSC and AM in the BALF of active TB patients. Results are given for the 16X (pink) and 20X (blue) pre-amplification cycles.

Supplementary Figure 3 ΔCt-values for all 43-genes assessed for differential expression between MDSC in the PB and BALF of active TB patients. Results are given for the 16X (pink) and 20X (blue) pre-amplification cycles.
