# Supplementary figures and images for: Targeted Gene Expression Profiling of Human Myeloid Cells From Blood and Lung Compartments of Patients With Tuberculosis and Other Lung Diseases
Source: Front Immunol. 2022 Mar 8;13:839747. doi: 10.3389/fimmu.2022.839747 (PMC8959218; doi:10.3389/fimmu.2022.839747)

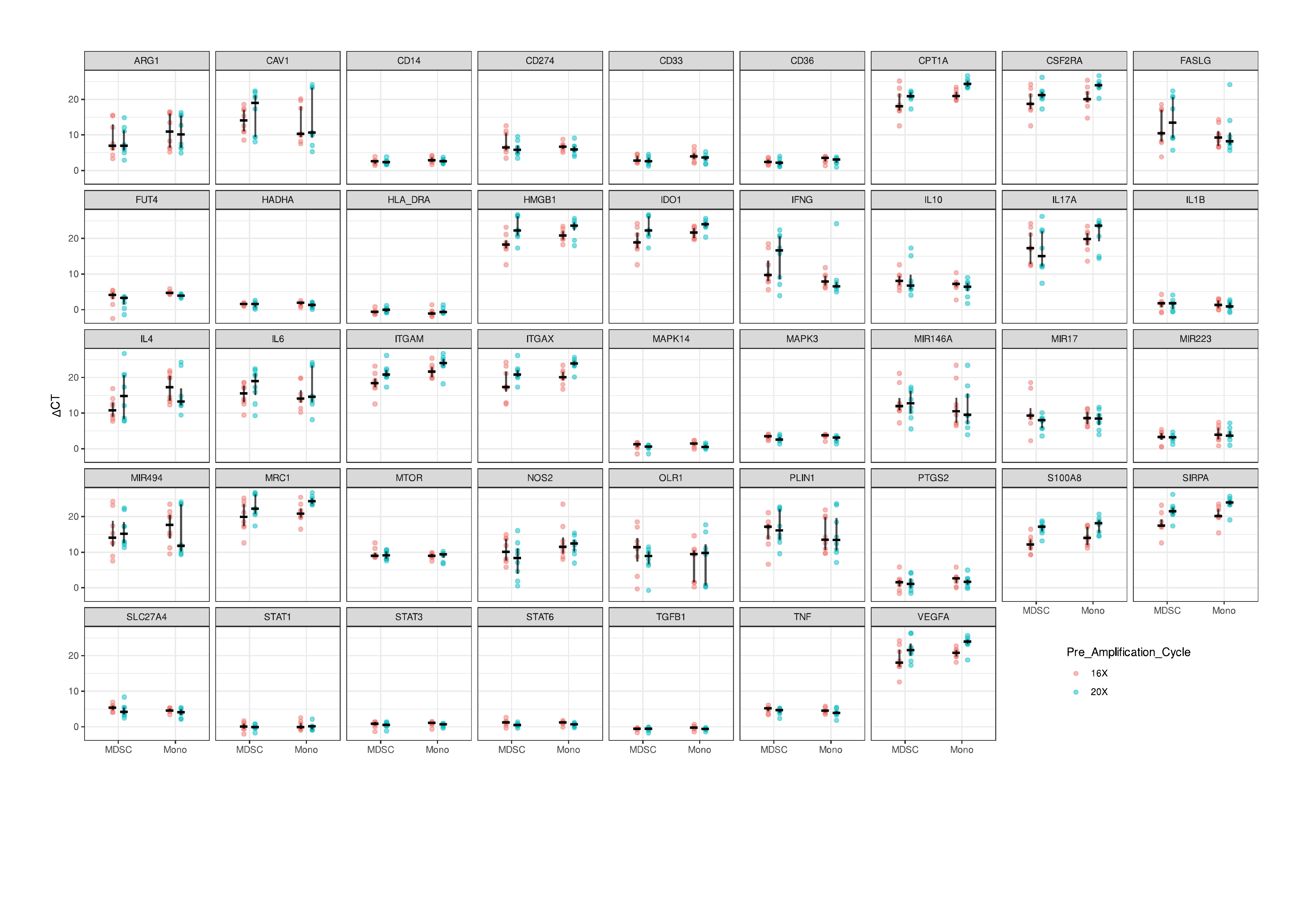

Supplement: Supplementary Figure 1 — ΔCt-values for all 43-genes assessed for differential expression between MDSC and monocytes in the PB of active TB patients. Results are given for the 16X (pink) and 20X (blue) pre-amplification cycles. [file Image_1.jpeg]

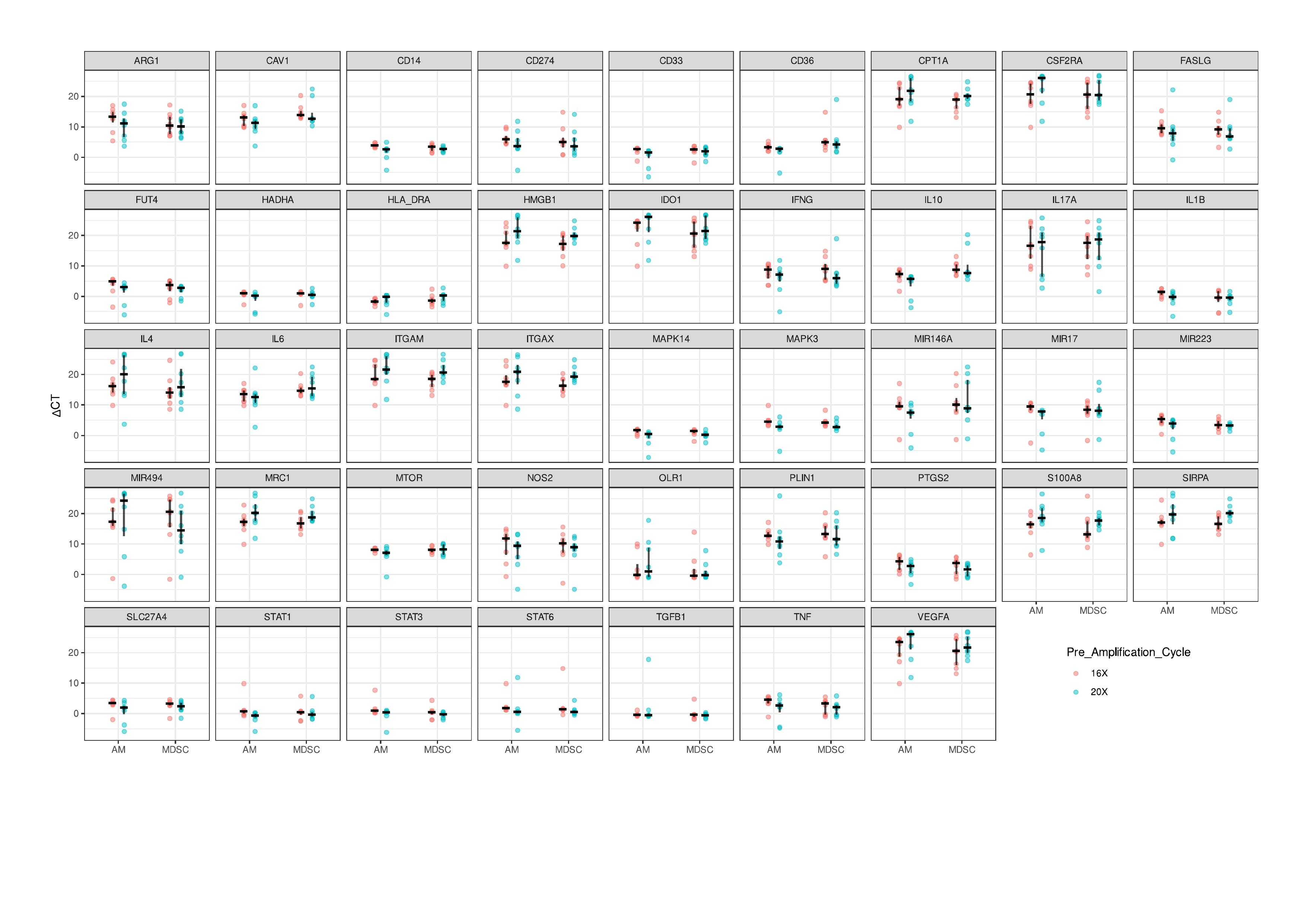

Supplement: Supplementary Figure 2 — ΔCt-values for all 43-genes assessed for differential expression between MDSC and AM in the BALF of active TB patients. Results are given for the 16X (pink) and 20X (blue) pre-amplification cycles. [file Image_2.jpeg]

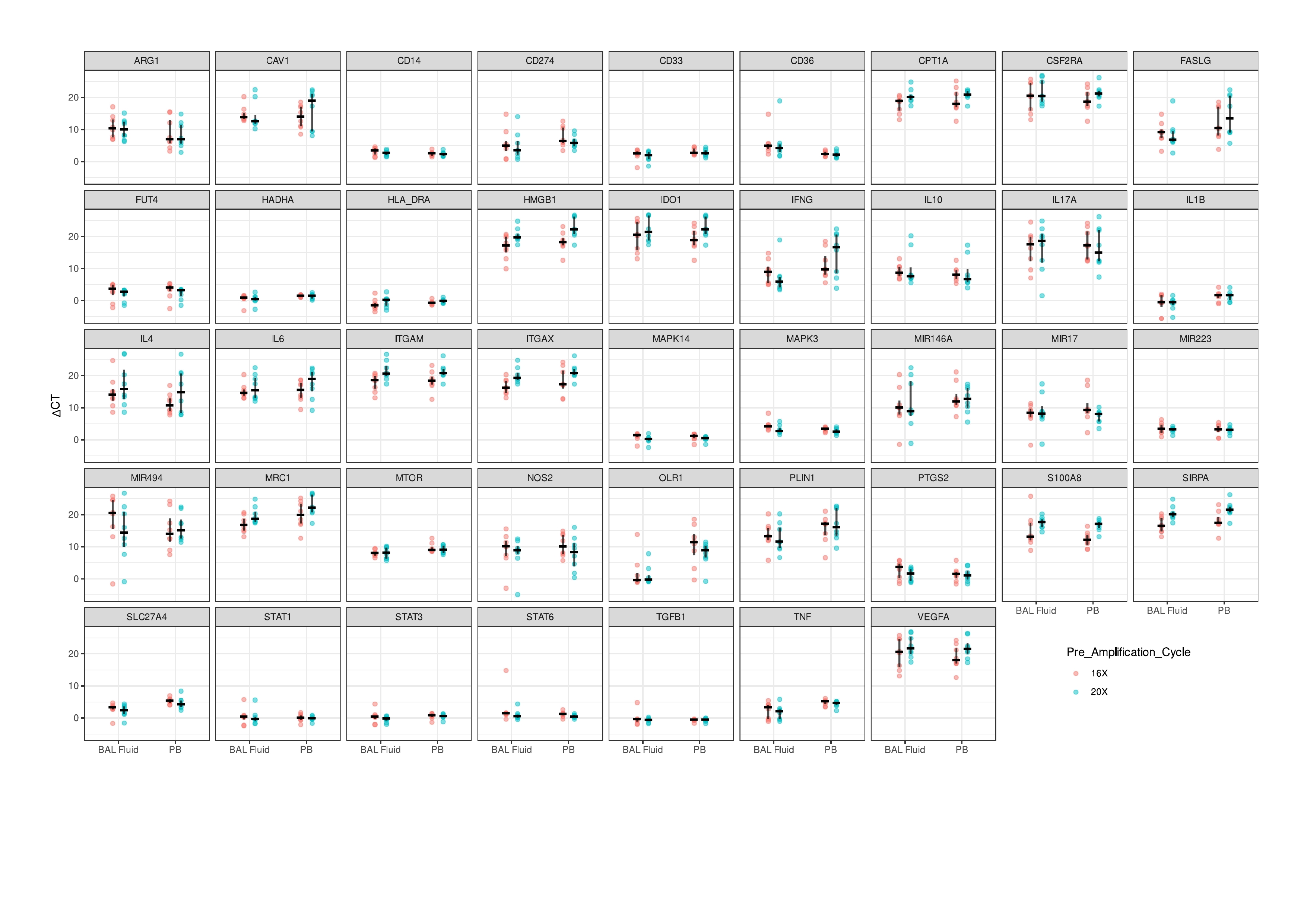

Supplement: Supplementary Figure 3 — ΔCt-values for all 43-genes assessed for differential expression between MDSC in the PB and BALF of active TB patients. Results are given for the 16X (pink) and 20X (blue) pre-amplification cycles. [file Image_3.jpeg]
