# Supplementary material for: Targeted Gene Expression Profiling of Human Myeloid Cells From Blood and Lung Compartments of Patients With Tuberculosis and Other Lung Diseases
Source: Front Immunol. 2022 Mar 8;13:839747. doi: 10.3389/fimmu.2022.839747 (PMC8959218; doi:10.3389/fimmu.2022.839747)
Supplement: Supplementary file 4 [file Table_1.docx]

Supplementary Table

Table S1: Correlation of all 43 genes expressed in MDSC between PB and BALF of active TB patients.

| Gene | Correlation | P-Value |
| --- | --- | --- |
| ARG1 | 0.029 | 0.917 |
| CAV1 | 0.212 | 0.43 |
| CD14 | 0.121 | 0.656 |
| CD274 | 0.306 | 0.249 |
| CD33 | 0.174 | 0.519 |
| CD36 | -0.297 | 0.263 |
| CPT1A | 0.209 | 0.436 |
| CSF2RA | 0.156 | 0.564 |
| FASLG | -0.65 | 0.008 |
| FUT4 | 0.124 | 0.648 |
| HADHA | 0.441 | 0.089 |
| HLA_DRA | 0.282 | 0.288 |
| HMGB1 | 0.259 | 0.332 |
| IDO1 | -0.009 | 0.978 |
| IFNG | -0.406 | 0.12 |
| IL10 | -0.232 | 0.385 |
| IL17A | -0.332 | 0.208 |
| IL1B | 0.294 | 0.268 |
| IL4 | 0.356 | 0.176 |
| IL6 | 0.235 | 0.379 |
| ITGAM | 0.359 | 0.173 |
| ITGAX | 0.044 | 0.874 |
| MAPK14 | 0.135 | 0.617 |
| MAPK3 | 0.215 | 0.423 |
| MIR146A | 0.306 | 0.249 |
| MIR17 | -0.279 | 0.294 |
| MIR223 | 0.091 | 0.738 |
| MIR494 | -0.335 | 0.204 |
| MRC1 | 0.029 | 0.917 |
| MTOR | -0.129 | 0.633 |
| NOS2 | -0.265 | 0.321 |
| OLR1 | -0.229 | 0.391 |
| PLIN1 | 0.388 | 0.138 |
| PTGS2 | 0.35 | 0.184 |
| S100A8 | 0.494 | 0.054 |
| SIRPA | 0.141 | 0.602 |
| SLC27A4 | 0.674 | 0.005 |
| STAT1 | 0.371 | 0.158 |
| STAT3 | 0.432 | 0.096 |
| STAT6 | 0.659 | 0.007 |
| TGFB1 | 0.488 | 0.057 |
| TNF | -0.256 | 0.338 |
| VEGFA | -0.156 | 0.564 |
